# Schwannoma of upper eyelid: A rare differential diagnosis of eyelid swellings

**DOI:** 10.4103/0970-0358.73456

**Published:** 2010

**Authors:** Surendra B. Patil, Satish M. Kale, Sumeet Jaiswal, Nishant Khare

**Affiliations:** Department of Plastic Surgery, Government Medical College, Nagpur, Maharashtra, India

**Keywords:** Eyelid tumours, malignant eyelid tumours, Schwannoma, S-100 protein

## Abstract

Schwannoma is a relatively rare benign tumour of peripheral nerve origin. The occurrence of Schwannoma in eyelid is extremely rare. As per our knowledge, only 11 such cases have been reported in the literature so far. We present a case of a 40-year-old man who presented to us with a 2-year history of slowly enlarging, painless mass in his left upper lid with resultant progressive ptosis. Ocular examination was suggestive of a firm, non-tender nodule of size 2 × 1.5 × 1 cm on the left upper lid. The mass was non-adherent to the skin or the underlying tissue. The eyelid skin and conjunctiva were indurated and signs of inflammation were present. The lateral part of eyelid showed presence of an ulcer and the lid function was severely hampered. Provisional clinical diagnosis was that of an eyelid malignancy. With this in mind, the medial part of the lid was excised and reconstructed using a tarso-conjunctival flap from the lower eyelid in conjunction with a skin graft. The histopathology and immunohistochemistry established the diagnosis of Schwannoma. We recommend that Schwannoma be considered in the differential diagnosis of well-circumscribed eyelid swellings.

## INTRODUCTION

Schwannoma is a relatively rare benign tumour of peripheral nerve origin. In ophthalmic region it has been reported in relation to the orbit and infrequently to conjunctiva,[1] uveal tract[2] and sclera.[3] The occurrence of Schwannoma in eyelid is extremely rare. As per our knowledge, only 11 such cases have been reported in the literature so far.[4–13] The growing reach of oculoplastic surgery mandates that Plastic Surgeons should be aware of this tumour and entertain it’s possibility in the differential diagnosis of eyelid tumours.

## CASE REPORT

**Figure 1:**
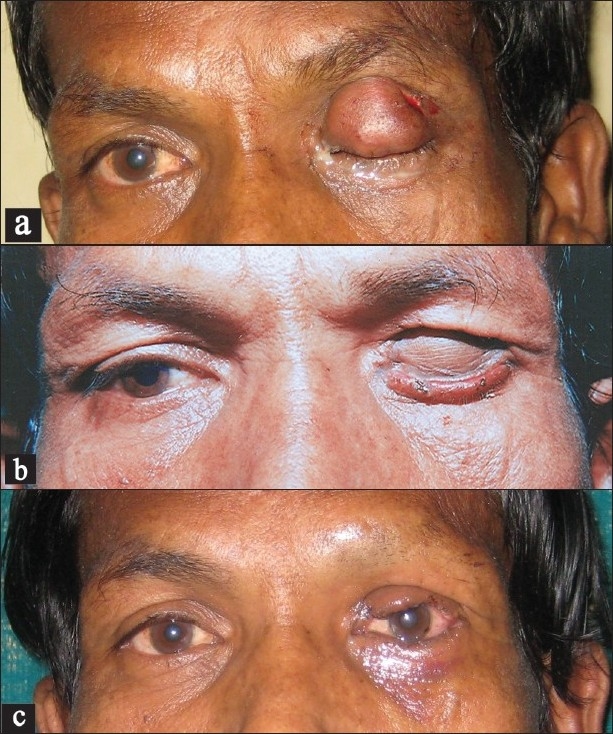
(a) Preoperative photograph showing swelling over the left upper eyelid; (b) intermediate photograph with flap *in situ*; (c) postoperative photograph

A 40-year-old man presented to us with a 2-year history of slowly enlarging, painless mass in his left upper lid with resultant progressive ptosis [Figure 1a]. The patient complained of constant sense of heaviness in the upper lid as well as partial ptosis. The superior field of vision was reduced. Ocular examination was suggestive of a firm, non-tender nodule of size 2 × 1.5 × 1 cm on the left upper lid. The mass was non-adherent to the skin or the underlying tissue. A small ulcer 5 mm × 8 mm was present on the lateral margin of the mass [Figure 1a]. The medial skin and conjunctiva were indurated and signs of inflammation were present. The lid appeared to have lost its function and was unsalvageable. Provisional clinical diagnosis was that of an eyelid malignancy. Fine needle aspiration cytology of the lesion was inconclusive.

The lesion was excised using a supratarsal incision. An ellipse was planned along the ulcer present on the lateral margin (0.8 mm) to excise the ulcer in continuity with the swelling. The lesion was 1.5 cm in diameter with a well-defined capsule, located below the subcutaneous tissue and above the muscle layer almost abutting the supraorbital rim. The incision was deepened and the lesion was isolated from the surrounding tissue by blunt dissection outside the capsule. Dissection was easily done in the extracapsular plane and the lesion was excised completely. No communication with the supraorbital nerve could be identified. However, the eyelid had strong evidence of induration and oedema, though no evidence of infiltration by the lesion was seen. As diagnosis of malignancy was suspected initially, the medial portion except for a small lateral margin was excised to ensure clear margins and reconstruction was done using a tarso-conjunctival flap from the lower eyelid in conjunction with a skin graft harvested from the left medial arm [Figure 1b]. The lateral rim of eyelid was closed primarily in continuity with the flap. The flap was divided at 3 weeks interval and the postoperative course was uneventful [Figure 1c].

Histologically, the tumour was formed by fusiform cells arranged in interwined bundles. The nuclei were fusiform and tended to form palisades. No histopathological features of malignancy were present. Immunohistochemistry for S-100 protein was strongly positive. These findings established the diagnosis of Schwannoma. Histopathology of the excised lid was suggestive of inflammatory changes. The patient recovered uneventfully and the ptosis resolved after ablation of the tumour with the improvement perceptible in the immediate postoperative period. There is no evidence of recurrence after 1 year of follow up.

## DISCUSSION

Schwannoma is rare benign neurogenic tumour made up of proliferating schwann cells of peripheral nerve. It is a neoplasm which occurs wherever schwann cells are present, that is, in any myelinated peripheral nerve. In most cases, while Schwannoma is sporadically manifested as a single benign neoplasm, the presence of multiple Schwannoma is usually indicative of neurofibromatosis-2. Our patient had isolated eyelid Schwannoma with no family history or clinical findings of neurofibromatosis-1 or neurofibromatosis-2.

Clinically, the tumour is a solid, slowly progressive and painless mass. Due to its rarity and unusual location, eyelid Schwannoma is frequently confused with other diagnosis like chalazion or inclusion cyst. In our case, patient presented with solid slowly enlarging mass of 2 years duration, started in middle third of left upper eyelid and later involving most of the eyelid. The tumour probably arose from branches of the supra orbital nerve. Literature suggests that the tumour, though rare, can be present in both upper and the lower eyelids. The size of the tumour ranges from few millimeters to 3.5 cm.[13] Management of Schwannoma of the eyelid is complete excision with clear margin to establish the histopathological diagnosis and prevent recurrence. Incomplete removal is associated with eventual recurrence and more aggressive behaviour. There have been anecdotal reports of malignant changes in a previously incompletely excised benign Schwanomma. The swelling also tends to transgress tissue planes and grow rapidly on incomplete excision.[5,6] An attempt to preserve continuity of nerve should be made, but this is not always possible and does not appear to have any major consequences at this site. Our case had a complete local excision and no evidence of recurrence exists on 1 year follow up.

## CONCLUSION

Accurate histological diagnosis and early complete excision of tumour should be the objective in managing Schwannoma of the eyelid. We recommend that Schwanomma be considered in the differential diagnosis of all well-circumscribed eyelid swellings. Lid salvage should be the goal of treatment, wherever possible, even in long standing disease.
